# Rotational landmarks of the distal femur in Indian population: A MRI-based study

**DOI:** 10.1186/s13018-015-0333-2

**Published:** 2015-12-21

**Authors:** Sivashanmugam Raju, Karthikeyan Chinnakkannu, Ramanivas Sunderayan, Mohan K. Puttaswamy

**Affiliations:** WF University School of Medicine, 241 S Cherry Street, # 227, Winston-Salem, NC 27101 USA; Shri Sathya Sai Medical College and Research institute, GT Main Road, Ammapettai, Tamil Nadu India; Apollo Specialty Hospital, Trichy, Tamil Nadu India; IORI Clinic, Bangalore, India

**Keywords:** Rotational landmarks, Condylar twist angle, Posterior condylar angle, Knee, Indian, Arthroplasty

## Abstract

**Background:**

Femoral rotational landmarks may vary according to the population. Our aim is to find out the relationship of the landmarks used in total knee arthroplasty in an Indian population and compare it with reported landmarks in other ethnic populations.

**Materials and methods:**

We retrospectively reviewed MR images of 124 knees in 124 patients to determine the relationship of bony landmarks by measuring the condylar twist angle (CTA), Whiteside-posterior condylar angle (W-PC), and Whiteside-epicondylar angle (W-EP). The difference between the genders and the sides was analyzed.

**Results:**

The mean CTA, W-EP and W-PC were 5.92°, 88.99° and 94.09° respectively. The mean CTA, W-EP and W-PC in males were 5.77°, 89.16° and 94.22° and they were 6.24°, 88.61° and 93.82° in females. On the left side, the CTA, W-EP and W-PC were 5.90°, 89.37° and 94.45° while they were 5.93°, 88.65° and 93.73° on the right side. There was no statistically significant difference between the genders or the sides.

**Conclusion:**

The CTA was around 6° in our study, and the posterior condylar angle (PCA) would be 3° as the difference between them is 3°. Hence, we conclude that the conventional jigs used in the measured resection technique using 3° external rotation in reference to the posterior condyles are still an appropriate option in normal and varus knees. And there is no difference between Indians and Caucasians, but there was a significant difference with Chinese populations. Although determining rotation based on the posterior condylar axis is more practical, it is prudent to combine it with other methods.

## Background

Rotational mal-alignment of the femoral component in total knee arthroplasty (TKA) impacts the outcome by requiring early revision [1–3]. Correct rotational alignment is necessary for normal patellar tracking and knee stability [4, 5]. Even a minor degree (1°–4°) of internal malrotation can cause lateral tracking and tilting [6]. Excessive external rotation may lead to flexion instability [7] and mechanical overload on the medial side of the joint [8]. The commonly used anatomical landmarks in achieving correct rotational alignment of the femoral component during surgery are the posterior condylar axis [5], anteroposterior axis [9], and transepicondylar axis [10, 11].

In India, TKA is done with data which has been derived from Western studies and there could be ethnicity-based differences which have not been studied yet in detail. But studies in other ethnic groups have reported that there was a definite anatomical difference between Japanese, Chinese, and Caucasians [12–14]. We have done a MRI-based study to determine the normal values of the variables like condylar twist angle (CTA), Whiteside-epicondylar angle (W-EP), and Whiteside-posterior condylar angle (W-PC) in an Indian population. The purpose of the study was to find out the relationship of the landmarks used in TKA in an Indian population and compare it with other populations to find out the modifications required when performing total knee replacement. Our hypothesis was that rotational landmarks in the Indian population would be different from those of the Caucasian population.

## Materials and methods

We retrospectively reviewed non-arthritic MR images of 124 knees of 124 patients stored in the diagnostic center system after obtaining approval from the Precision Diagnostics, India institutional review board. There were 124 patients including 86 males and 38 females, and the average age was 33.7 years (range 19 to 60 years). Our inclusion criterion was any patient who had undergone MRI for posttraumatic evaluation. Any patient with cartilage damage or any form of degenerative changes was excluded from the study.

**Fig. 1:**
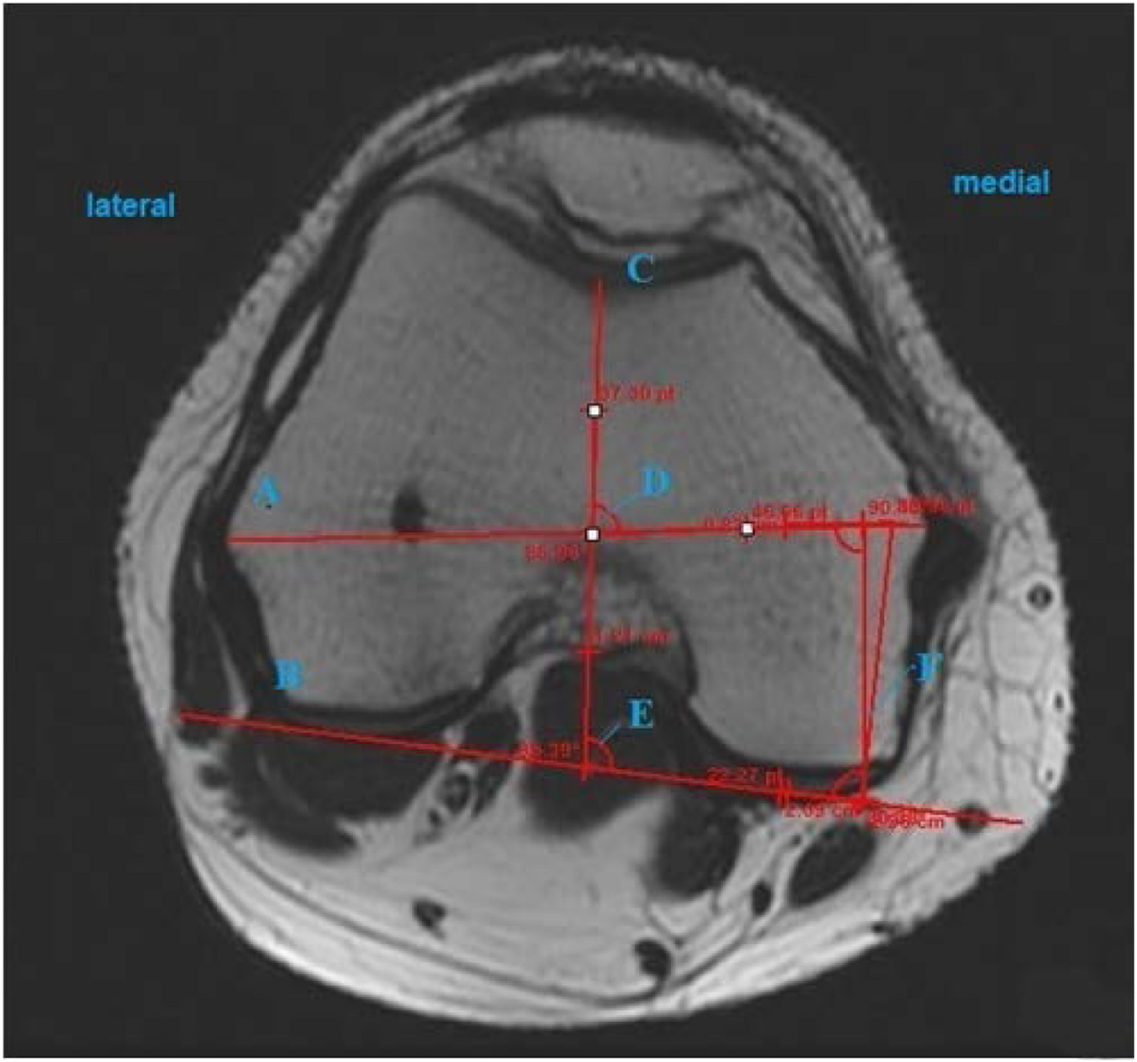
MRI of the distal femur with the axes and angles. *A* Clinical epicondylar axis, *B* posterior condylar axis, *C* anteroposterior axis, *D* Whiteside-epicondylar angle, *E* Whiteside-posterior condylar angle, *F* condylar twist angle

MRI was done using the following: 1.5 T units and 4000/80 (TR/TE); field of view—16 cm^2^; matrix—224 × 288; 5-mm slice thickness with an interslice gap of 1 mm; and excitations 2. Axial T2 FS images were used to assess the transepicondylar axis (TEA), anteroposterior axis, and posterior condylar axis (Fig. 1). The surgical TEA is drawn from the medial epicondylar sulcus to the prominent point of the lateral epicondyle. The clinical TEA was measured between the most prominent points of the medial and lateral epicondyles. The anteroposterior axis is a line drawn from the deepest part of the trochlear sulcus to the center of the intercondylar notch. The posterior condylar axis is a tangent connecting the posterior most summits of the posterior condyles.

The condylar twist angle (CTA) is the angle between the clinical transepicondylar axis and posterior condylar axis [10, 15]. The posterior condylar angle (PCA) is the angle between the surgical epicondylar axis and posterior condylar axis [10]. Both have been used in clinical studies to analyze the component rotation, but Suter et al. have proved that CTA is more reproducible than PCA in their CT-based study [16]; hence, we have used the clinical transepicondylar axis in our study. Based on these axes, the CTA, Whiteside-epicondylar angle (W-EP), and Whiteside-posterior condylar angle (W-PC) were calculated.

Measurements were done by two independent observers. The interobserver correlation coefficient was 0.5 (*p* = 0.001), and the intraobserver correlation coefficient was 0.64 (*p* = 0.032). The difference between the genders and the sides was analyzed with independent *T* test. A *p* value of <0.05 was taken to be statistically significant. Statistical analysis was done using IBM SPSS Statistics for Windows, Version 20.0, Armonk, NY: IBM Corp.

## Results

The mean CTA, W-EP, and W-PC were 5.92° (SD 2.32; range 0–13), 88.99° (SD 2.86; range 81–94), and 94.09° (SD 2.84; range 86–99), respectively (Table 1). The mean CTA, W-EP, and W-PC in males were 5.77°, 89.16°, and 94.22°, respectively, and they were 6.24°, 88.61°, and 93.82° in females. On the left side, the CTA, W-EP, and W-PC were 5.90°, 89.37°, and 94.45°, respectively, while they were 5.93°, 88.65°, and 93.73°, respectively, on the right side. There is no statistically significant difference between the genders or the sides for all the parameters (Table 2).

**Table 1:** Descriptive statistics of the rotational landmarks

Parameters	Number of knees	Mean	Standard deviation	Range
CTA	124	5.92	2.32	0–13
W-EP	124	88.99	2.86	86–93
W-PC	124	94.09	2.84	86–99

**Table 2:** Rotational landmark values of genders and sides

Parameters	Gender/side	Number of subjects	Mean	Standard deviation	*p* value
	Gender				
CTA	Male	86	5.77	2.28	0.307
	Female	38	6.24	2.40	
W-EP	Male	86	89.16	2.78	0.318
	Female	38	88.61	3.03	
W-PC	Male	86	94.22	2.75	0.466
	Female	38	93.82	3.06	
	Side				
CTA	Left	62	5.90	2.37	0.95
	Right	62	5.93	2.29	
W-EP	Left	62	89.34	2.75	0.18
	Right	62	88.65	2.94	
W-PC	Left	62	94.45	2.96	0.15
	Right	62	93.73	2.68	

## Discussion

Correct rotational alignment of the distal femoral component is one of the determinants of good functional outcome following TKA. Excessive internal rotation results in patellar complications while excessive external rotation can cause flexion instability [4, 5]. To achieve the correct rotation, appropriate landmarks and angles should be used as they vary across populations [12, 13].

Studies determining the exact rotational position of the bony anatomy of the distal femur have usually employed three different methods. They are direct measurement with a goniometer either intraoperatively or in a cadaver and measurement using CT scan and MRI [11, 17–19]. Direct measurement is confounded by difficulty in determining the exact position of the sulcus in the medial epicondyle. During surgery, rotational alignment is measured with the cartilage in situ but many studies looking into the rotational alignment of the femoral component have employed CT scan, which does not give good assessment of the cartilage anatomy. We felt a MRI-based study would be more appropriate as the angles could vary based on the cartilage thickness [20, 21]. Moreover, during the surgery, the bony resections and component position are influenced by the cartilage thickness.

Among the epicondylar axes, the surgical epicondylar axis was proved to be representing the true rotational axis as the flexion-extension of the knee happens around this axis [22, 23]. But the medial sulcus could not be identified in nearly 50 % of the cases even on CT [15, 18]. Moreover, identifying the medial sulcus during surgery is even more challenging as it requires extensive dissection especially in an arthritic knee [14, 18]. Suter et al. have shown that the clinical epicondylar axis was more reproducible while using CT [16]. We faced the same problem of identifying the sulcus and calculating the posterior condylar angle (PCA); hence, we used the clinical epicondylar axis and measured the condylar twist angle (CTA).

In our study, the mean CTA was 5.92° (SD 2.32) with a variation of 5.78° in males and 6.24° in females. But the difference is statistically not significant. In 1987, Yoshioka et al. were the first one to describe the CTA in a cadaveric study, and the CTA was 5° and 6° in males and females, respectively [24].

Our CTA is in concurrence with most of other cadaveric studies in a Caucasian population (Table 3). The CTA reported by most of them lies between 4.4° and 6.1° [9, 10, 24–26]. But Chinese knees appear to be more externally rotated as compared to Indian knees, and their adjusted CTA would be 8.5° based on their PCA values [13].

**Table 3:** Rotational axes and angles from various studies

Studies	Condylar twist angle (CTA)	Posterior condylar angle (PCA)	W-EP	W-PC
Cadaveric studies				
Arima et al. [9]	4.4 (2.9)			
Berger et al. [10]	Male 4.7 (3.5)	Male 3.5 (1.2)		
	Female 5.2 (4.1)	Female 0.3 (1.2)		
Yip et al. [12]		Males 5.1 (1.9)		
		Females 5.8 (1.8)		
Yoshioka et al. [24]	Male 5 (1.8)			
	Female 6 (2.4)			
Mantas et al. [25]	Right 4.9 (2.1)			
	Left 4.9 (2.3)			
	Male 4.4 (2)			
	Female 6.4 (2.2)			
Katz et al. [26]	6.1 (3.3)			
Intraoperative studies				
Poilvache et al. [14]	3.60 (2.02)		90.33 (2.44)	86.92 (2.71)
	Males 3.58 (2.16)		Male 91.2 (2.15)	Male 88.07 (2.34)
	Female 3.62 (1.93)		Female 89.59 (2.45)	Female 85.94 (2.64)
	Varus-neutral 3.51 (2.03)		Varus-neutral 90.53 (2.36)	Varus-neutral 87.27 (2.57)
	Valgus 4.41 (1.83)		Valgus 88.73 (2.57)	Valgus 84.09 (2.21)
Griffin et al. [11]		Average 3.7 (2.2)		
		Male 3.6 (1.8)		
		Female 3.7 (2.6)		
		Varus 3.3 (1.9)		
		Neutral 3.3 (2.3)		
		Valgus 5.4 (2.3)		
Radiograph-based studies				
Arima et al. [9]	5.7 (1.7)			
CT-based studies				
Akagi et al. [5]	OA knees 6.8 (1.8)			
Nagamine et al. [27]	Normal 5.8 (2.7)		87.7 (3.9)	93.5 (4)
	PF-OA 6.4 (2.4)			
	FT-OA 6.2 (1.9) (arthritic knees)			
Yoshino et al. [18]	6.4 (1.6) (arthritic knees)	Average 3 (1.6)		
Takai et al. [28]	6.8 (2) (arthritic knees)			
	6.3 (1.5) (normal knees)			
Mullaji et al. [30]	5 (1.7) (normal knees)		90.8 (3.7)	95.8 (3.5)
MRI-based studies				
Current study	5.92 (2.32) (normal knees)		88.99 (2.86)	94.09 (2.84)
Matsuda et al. [19]	Normal 6.03 (3.60)			
	Varus 6 (2.35)			
Griffin et al. [11]		3.11 (1.75)		
		Male 2.75 (1.61)		
		Female 3.33 (1.82)		
		<41 years 2.71 (1.56)		
		>41 years 3.50 (1.86)		

Mean values are given in degrees (°). Those in brackets are the standard deviation

Our result demonstrates increased CTA compared to that of Poilvache et al. (5.92° vs. 3.6°), and the difference could be possibly due to difficulty in determining the exact position of the medial sulcus intraoperatively [14]. It is less likely to represent any racial difference (Indian vs. Caucasian) as other cadaveric studies in Caucasians have shown a similar CTA.

CT-based studies (Table 3) were done in both normal and arthritic knees [27, 28]. Previous studies in Japanese knees found that there was not any significant difference in CTA between normal and arthritic varus knees [27, 28] as the posterior femoral condyles were well preserved till the end even in arthritic knees [29]. CT-based results in the Japanese population were close to our results and concur with another MRI-based study in a Japanese population as well [19].

Our CTA is slightly higher as compared to that of a previous Indian study [30], but there is no significant difference compared to other MRI-based studies (Table 3). Overall, comparing the CT- and MRI-based studies, CTA value is slightly more in MRI-based studies. This phenomenon may be due to the difference in the cartilage thickness and concurs with Tashiro et al.’s observation [20].

In a previous CT-based study by Mullaji et al. in normal Indian knees, the PCA was 5° [30] but they did not differentiate between the CTA and PCA and they suggested 2° additional of external rotation when using the posterior condyles as reference to avoid internal rotation of the femoral implant. A study by Katz et al. [26] also failed to differentiate them. There is a definite confusion in literature in differentiating the two, and we also believe that most surgeons use epicondylar prominence for component rotation which is essentially the CTA.

Measured resection is the most popular technique in India, and the conventional jigs have 3° of inbuilt external rotation. Failure to make the difference between the CTA and PCA would lead to component position in additional 3° of external rotation along with 3° inbuilt external rotation. Even though only few studies emphasized the effects of excessive external rotation, it leads to more bone resection from the medial side, medial overloading, and medial instability [2, 7, 8]. We do not have any long-term clinical studies which specifically looked at minor degrees (3°–6°) of excessive external rotation, and there is no clear cutoff value for that. Further studies are warranted to find out what constitute excessive based on ethnicity and long-term effects of minor degrees of excessive external rotation.

Yoshino et al. in their CT-based study have stated that PCA can be calculated from the condylar twist angle by subtracting 3° [18], and this view was reiterated by Akagi et al. and they proved that the relation between the CTA and PCA was a constant 3° of external rotation if the femoral valgus angle is less than 9° [15]. In that case, our PCA would be approximately 3°, which is consistent with the other studies. So, it is more appropriate to use a combination of 3° external rotation and clinical epicondylar axis.

The mean W-PC was 94.09° (externally rotated), and the mean W-EP was 88.99° (internally rotated). There was no statistically significant difference between the genders and sides. The W-PC value indicates that the component has to be 4° externally rotated to the posterior condylar axis to match the Whiteside line. Based on the W-EP values, the femoral component needs to be parallel or slightly internally rotated to the clinical epicondylar axis to align with the Whiteside line. These values are approximately close to those reported by Arima et al. and Katz et al. in which the W-PC was 93.1° (SD 1.7) and 93.4°, respectively [9, 26]. But reproducing the exact alignment during surgery is still difficult as intraoperative errors are common [31, 32]. Poilvache et al.’s intraoperative measurement in Caucasians which resulted in W-PC and W-EP of 86.92° and 90.33°, respectively, supports this [14].

The limitation of our study is that we have not measured the mechanical axis of the lower extremity. We have taken the difference of 3° between the CTA and PCA which was studied in the Caucasian population, and we have not measured the difference between the PCA and CTA in the Indian population.

The surgical epicondylar axis represents the true rotational axis of the knee; hence, determining rotation using the surgical epicondylar axis is the best method, but intraoperative errors are common as it is difficult to identify the medial sulcus in an arthritic knee. Moreover, intraoperatively, it is easy to identify the epicondylar prominences but the clinical epicondylar axis is 3° more externally rotated in relation to the surgical epicondylar axis, and failure to make this difference would lead to more external rotation (around 3°) than required. So, it is rather more practical and reproducible to use the posterior condylar axis to determine the rotation.

## Conclusions

From our study, the CTA is around 6° and the PCA would be 3° as the difference between them is 3°. Hence, we conclude that the conventional jigs used in the measured resection technique using 3° external rotation in reference to the posterior condyles are still an appropriate option in normal and varus knees. And there is no difference between Indians and Caucasians, but there was a significant difference with Chinese populations. Although determining rotation based on the posterior condylar axis is more practical, it is prudent to combine it with other methods.

## Abbreviations

CTA: condylar twist angle
PCA: posterior condylar angle
TEA: transepicondylar axis
TKA: total knee arthroplasty
W-EP: Whiteside-epicondylar angle
W-PC: Whiteside-posterior condylar angle
